# Regulation and Implementation of Apoptosis in Melanoma Tumor Cells with *BRAF*, *NRAS*, and *NF1* Gene Mutations

**DOI:** 10.3390/cimb48050434

**Published:** 2026-04-22

**Authors:** Olga L. Nosareva, Ekaterina A. Pomytkina, Olga N. Korotkova, Alexandra V. Tumanova, Elena A. Stepovaya, Vladimir M. Nagaitsev, Elena A. Kochurina, Olga V. Bakina, Oleg V. Kokorev, Liudmila V. Spirina

**Affiliations:** 1 Biochemistry and Molecular Biology Department, Siberian State Medical University, 2, Moskovsky Trakt, Tomsk 634050, Russia; olnosareva@yandex.ru (O.L.N.); katyapomytkina2004@gmail.com (E.A.P.); olgaok5171@gmail.com (O.N.K.); alextu761@gmail.com (A.V.T.); stepovaya.ea@ssmu.ru (E.A.S.); ovbakina@ispms.ru (O.V.B.); kokorevov@yandex.ru (O.V.K.); 2 Altajskij Kraevoj Onkologicheskij Dispenser, Zmeinogorski Trakt, 110, Barnaul 656045, Russia; vn71@list.ru; 3 Moscow State Budgetary Healthcare Institution “Moscow Multidisciplinary Clinical Center “Kommunarka” of the Moscow City Department of Healthcare”, Intra-City Municipality of Sosenskoye Settlement, Kommunarka Settlement, 8, Building 3, Sosensky Stan Street, Moscow 108814, Russia; kochurina.elena@mail.ru

**Keywords:** apoptosis, human melanoma, cell redox-status, oxidative stress, gene mutation *BRAF*, gene mutation *NRAS*, gene mutation *NF1*

## Abstract

This review explores the molecular mechanisms regulating signaling pathways that may be potentially involved in modulating apoptosis in melanoma tumor cells harboring mutations in *BRAF*, *NRAS*, and *NF1* genes. The high mortality associated with the aggressive nature of melanoma, its significant metastatic potential, and resistance to existing cancer therapies underscore the relevance of this research topic. One contributing factor to the poor therapeutic prognosis is the altered activity of signaling pathway components that promote the survival of melanoma cells. The increased invasion and metastasis of melanoma cells are linked to mutations in *BRAF*, *NRAS*, and *NF1* genes. Under conditions of oxidative stress within tumor cells, oxidative modifications occur in proteins that are part of these signaling pathways. Reversibly and irreversibly oxidized proteins, including components of signaling pathways, represent molecular targets for the regulation of apoptosis in melanoma tumor cells. Elucidating the molecular mechanisms underlying the regulation of apoptosis in tumor cells harboring mutations enables the proposal of potential molecular approaches for targeted therapy of melanoma.

## 1. Introduction

In the pathogenesis of tumor growth, malignant cells are the critical component. In these cells, the modulation of signaling pathways is accompanied by the activation of molecular mechanisms related to proliferation, evasion from immune surveillance, and cell death. During transformation, tumor cells acquire the ability to invade and metastasize. To support these processes, tumor cells require a reorganization of metabolism, particularly in ATP, synthesis, protein synthesis, and glucose oxidation [1,2,3]. Tumor cells with genomic mutations exhibit distinct regulatory features in their metabolic pathways. An example of malignancy associated with the evasion of apoptosis by tumor cells in the presence of genomic mutations is skin melanoma [4,5].

Skin melanoma is one of the most aggressive forms of skin cancer [6,7,8,9]. It is a malignant tumor of neuroectodermal origin derived from skin melanocytes. Melanoma is characterized as a rapidly progressing cancer type that shows resistance to chemotherapy, radiation therapy, and immunotherapy. One reason for the unfavorable therapeutic prognosis is the activation of signaling pathways that enhance tumor cell survival [5,7,10,11]. The incidence of melanoma has increased significantly due to heightened exposure of the skin to ultraviolet radiation [7,8,12,13]. Key molecular factors contributing to increased invasion and metastasis include mutations in the *BRAF*, *NRAS*, and *NF1* genes in melanoma tumor cells [5,8,14,15].

Melanoma is one of the most metastatic types of cancer in humans. It has been established that a primary melanoma tumor, containing approximately one million cells, can have a significant metastatic potential compared to most solid tumors, which typically contain around one trillion cells [12]. Studying the molecular mechanisms by which genomic mutations affect the metabolism and apoptosis of tumor cells can help to identify markers for targeted therapy in skin melanoma. For instance, immune checkpoint inhibitors targeting PD-1, CTLA-4, and LAG-3 are effective for patients with both *BRAF* mutations and wild-type melanoma. These checkpoint inhibitors have shown justified results in treating patients, despite some associated immune-related side effects [7,16,17,18]. Analyzing these findings suggests that the unique metabolism of tumor cells may influence melanoma treatment, contribute to drug resistance, and enhance tumor cell survival.

Research focused on understanding the metabolic characteristics of tumor cells, followed by identification of potential markers for early diagnosis and monitoring of melanoma treatment, remains highly relevant. The increasing incidence of melanoma, the aggressive nature of the disease, and the need for new prevention and treatment strategies underscore the demand for studies examining metabolic changes, regulatory pathways, and mechanisms of cell death during melanocyte malignancy. A critical aspect of tumor transformation in melanocytes under oxidative stress is the presence of mutations in these cells. Oxidative stress alters the levels of oxidatively modified macromolecules, leading to changes in metabolism as well as the regulation and execution of apoptosis in malignant melanocytes [19,20,21,22,23,24,25]. Modulation of protein conformation represents a molecular technology for regulating protein activity and intracellular biochemical processes [26,27,28,29]. Of particular interest is the potential to influence the functional state of protein components within signaling cascades through reversible and irreversible oxidative modification of proteins, with the aim of enhancing the sensitivity of melanoma cells to antitumor therapy. Therefore, one of the key approaches to molecularly managing cell death under oxidative stress is the redox modulation of signaling pathway components, particularly in melanoma cells with mutations.

## 2. Regulation and Execution of Cell Apoptosis

Apoptosis is an active form of cell death, serving as a physiological mechanism for eliminating cells that are functionally deficient or defective in terms of receptor representation. The phenomenon of apoptosis arises from various factors that lead to cell death [11,30,31]. Apoptosis can be divided into three phases: initiation, apoptosis development or the effector phase, and degradation. During the degradation phase, proteins and DNA undergo hydrolysis with complete reorganization of the cytoskeleton. Currently, it is common to distinguish between membrane, mitochondrial, and nuclear mechanisms of apoptosis induction. The development of the effector phase is influenced by the signal transduction initiation mechanism, which can be external (receptor-dependent signaling pathways (Tumor Necrosis Factor (TNF) superfamily, TNF receptor type I (Tumor Necrosis Factor Receptor I Type, TNF RI)) or internal (the mitochondrial pathway of cell death) [31,32]. Dysregulation of the cell death program can lead to pathological changes in organs and tissues, disrupting their functions. The activation or inhibition of apoptosis is fundamental to the pathogenesis of socially significant diseases such as cancer, cardiovascular, neurodegenerative, and endocrine diseases; inflammatory processes of both infectious and non-infectious origins; etc. [30,33]. For instance, an imbalance between pro-apoptotic and anti-apoptotic factors that inhibits programmed cell death is a primary cause of tumor transformation in normal cells and the malignancy of benign tumors.

Various components of signaling systems, such as RAS/MAPK-, PI3K-AKT-, and cAMP pathways, play crucial roles in executing apoptotic pathways [34,35,36,37].

The role of the RAS/MAPK signaling cascade in apoptosis is context-dependent. This pathway is a regulatory module that can either suppress or enhance apoptosis. Its primary function is to transmit signals from external stimuli that promote cell proliferation, differentiation, and survival. However, under certain conditions, it can become pro-apoptotic. External stimuli may include growth factors (Epidermal Growth Factor (EGF); Nerve Growth Factor (NGF); Vascular Endothelial Growth Factor (VEGF)), mitogens, and hormones [38]. When this pathway is activated, receptors with tyrosine kinase activity dimerize and autophosphorylate. Proteins Grb2 (Growth Factor Receptor Bound Protein 2) and Shc (Src homology 2 domain-containing transforming protein 1) bind to the phosphorylated tyrosine on the receptor. This leads to the activation of the RAS: Grb2 recruits a GEF (Guanine Nucleotide Exchange Factor), which exchanges GDP for GTP in the small G-protein RAS (K-Ras, H-Ras, and N-Ras), resulting in an active GTP-bound form. RAS-GTP activates the serine/threonine kinase RAF (C-RAF, B-RAF). RAF phosphorylates and activates MEK1/2 kinases (MAPK/ERK kinase). MEK1/2 phosphorylate ERK1/2 (Extracellular signal-Regulated Kinases) kinases at tryptophan and tyrosine residues. Active ERK1/2 then phosphorylates numerous cytoplasmic and nuclear targets (specific proteins such as transcription factors, other kinases, and ribosomal proteins), leading to modulation of cell proliferation, differentiation, and survival [34,38].

The primary role of the RAS/MAPK pathway is anti-apoptotic. This pathway directly opposes the initiation of both external (receptor-mediated) and internal (mitochondrial) apoptosis. The suppression of the external (receptor) pathway occurs through the phosphorylation and inactivation of caspase-8 and caspase-10 (ERK can phosphorylate procaspase-8, preventing its recruitment to the DISC (Death-Inducing Signaling Complex) and activation). The suppression of the internal (mitochondrial) pathway is associated with the phosphorylation and degradation of Bim (a potent pro-apoptotic BH3 protein that activates Bax (BCL2 Associated X, Apoptosis Regulator)/Bak (BCL2 Antagonist/Killer); ERK phosphorylates Bim, marking it for ubiquitin-dependent proteasomal degradation) and Bad (BH3 protein that, when phosphorylated by ERK and AKT, cannot inhibit Bcl-2/Bcl-XL) [32].

In cases where anti-apoptotic transcriptional programs are enhanced, ERK phosphorylates and activates transcription factors such as c-Fos, c-Myc, and CREB. These factors induce the expression of anti-apoptotic genes of proteins: Bcl-2 and Bcl-XL, both inhibitors of Bax/Bak; c-FLIP, a homolog of caspase-8 without catalytic activity that competes with caspase-8 for binding to FADD (Fas Associated Death Domain) in the DISC, thus blocking the external pathway; the IAP family (e.g., Survivin), caspase inhibitors; and Mcl-1, a short-lived anti-apoptotic protein from the Bcl-2 family, critical for the survival of many cells. In addition, through interaction with the PI3K-AKT signaling pathway, RAS can directly activate PI3K, enhancing survival signals in tumor cells [32].

Thus, activation of the RAS/MAPK pathway raises the threshold of cell sensitivity to apoptotic stimuli. This is why oncogenic mutations in RAS or BRAF (such as those found in melanoma) lead not only to uncontrolled proliferation but also to resistance of tumor cells to apoptosis and chemotherapy.

Under certain conditions, the RAS/MAPK signaling pathway can promote cell death. For instance, during excessive or prolonged activation of the pathway or in the absence of parallel pro-apoptotic signals (such as from PI3K/AKT). The pro-apoptotic mechanisms of the RAS/MAPK pathway involve inducing the expression of pro-apoptotic proteins. Activated transcription factors (e.g., c-Myc) can induce pro-apoptotic genes such as *BAX*, *NOXA*, and *PUMA*. If parallel survival signals are weak, apoptotic cell death occurs [34].

Cells with hyperactive RAS exist in a state of “apoptosis readiness” due to increased expression of pro-apoptotic proteins (e.g., Bim, Puma, and Noxa). However, they can survive through compensatory activation of parallel survival pathways (PI3K-AKT and transcription of anti-apoptotic proteins). When these compensatory pathways are inhibited, cells with mutant RAS quickly undergo apoptosis.

The RAS/MAPK signaling pathway integrates effects that determine cell fate (life or death), influenced by other signaling pathways in terms of timing and strength of component activation.

In summary, various mechanisms for regulating apoptosis pathways in tumor cells exist. In our view, a promising molecular approach is the redox-based regulation of signaling pathway components, which are proteins.

## 3. Regulation and Implementation of Apoptotic Cell Death in Melanoma Cells with Genomic Mutations

The malignant transformation of melanocytes occurs due to mutations in genes of proteins—components of signaling pathways that are critical for cell survival. One such pathway involves mitogen-activated protein kinases (MAPKs), which play a role in various physiological processes, including proliferation, differentiation, migration, apoptosis, and cell transformation. Disruption of the function of these kinases is crucial for the development of melanoma. Malignant melanocytes are characterized by the hyperactivation of mitogen-activated protein kinase 1 (MAP2K1) and extracellular signal-regulated kinases (ERK), which are activated by external signals. This activation subsequently triggers transcription factors responsible for regulating and executing cell proliferation and death [39].

Another signaling cascade involved in the regulation of transcription, translation, differentiation, proliferation, and increased survival is the PI3K-AKT pathway. This pathway includes the activation of phosphatidylinositol 3-kinase (PI3K), related receptor tyrosine kinases, and G-protein-coupled receptors (GPCR). The activation of phosphatidylinositol 3-kinase (PI3K) leads to the production of phosphatidylinositol 3,4,5-trisphosphate (PIP3) through the phosphorylation of phosphatidylinositol 4,5-bisphosphate (PIP2) at the plasma membrane. Phosphatidylinositol 3,4,5-trisphosphate (PIP3) is necessary for enhancing the affinity of serine-threonine protein kinase (AKT) for lipids of the plasma membrane. Then AKT is involved in phosphorylation of various specific proteins that are its substrates. This results in the inhibition or activation of specific proteins and the regulation of important cellular processes such as glucose metabolism, apoptosis, proliferation, invasion, and angiogenesis [16,36,40,41]. In addition, AKT inhibits pro-apoptotic proteins, including BAD and caspase-9, as well as Forkhead Box O (FOXO) transcription factors that regulate transcription, proliferation, and cell survival [42].

In melanocytes, besides the RAS/MAPK signaling pathway, the cAMP signaling cascade also plays a significant role [19]. Activation of this pathway by various ligands increases cAMP levels in melanocytes, leading to the activation of protein kinase A (PKA), which phosphorylates CREB. This process subsequently activates the transcription factor MITF, which is responsible for melanocyte differentiation. The MITF transcription factor regulates the expression of genes (*TYR*, *TYRP1*, *DCT*, *RAB27A*, and *GPR143*) associated with melanin synthesis and melanosome function [4]. The intracellular levels of cAMP are regulated by phosphodiesterases (PDEs), whose activity is modulated by specific protein kinases. This positions PDEs as regulators of the interaction between the cAMP pathway and other intracellular signaling pathways. Under physiological conditions, the MAPK pathway is activated through receptor tyrosine kinases (RTK). However, constitutive activation of the cAMP pathway in melanocytes leads to the phosphorylation and inactivation of c-RAF by PKA. Consequently, RTK activation stimulates the MAPK pathway in melanocytes via *BRAF*. Mutations in the *BRAF* and *NRAS* genes in melanoma cells are associated with the activation of the RAS/MAPK signaling pathway [15,18].

In a study conducted by Chen X.Y. et al. [10] on A375 tumor cell lines, the anti-proliferative and pro-apoptotic effects of vitamin C were demonstrated, which were associated with the activation of caspase-9 and caspase-3 alongside a significant reduction in mitochondrial potential [10]. Notably, it was the reduced form of ascorbic acid that exhibited these pro-apoptotic and anti-proliferative effects. Reduced glutathione plays a crucial role in maintaining the reduced form of ascorbate, as it directly participates in the redox reactions of tumor cells [10]. From this, it can be inferred that the levels of reduced vitamin C may influence the redox status of tumor cells, which is involved in regulating apoptosis.

The PI3K-AKT-mTOR signaling pathway is involved in the regulation of apoptotic cell death in melanoma cells. The PTEN-PI3K-AKT pathway is negatively regulated by PTEN (Phosphatase and Tensin Homolog Deleted on Chromosome 10), an inhibitor of the PI3K kinase that has phosphatase activity. PTEN acts as a tumor growth suppressor. Inactivation of PTEN promotes the survival of tumor cells by inhibiting pro-apoptotic signaling through the activation of serine/threonine protein kinase (AKT) and agonists of cell death associated with the *BCL2* gene family (BAD) [36]. The tumor suppressor gene *PTEN* is frequently mutated in melanoma cells, following mutations in *BRAF* and *NRAS*, contributing to cell survival [18,42].

Phosphoinositide 3-kinases (PI3K) are members of a family of intracellular lipid kinases that phosphorylate the 3′-hydroxyl group of phosphatidylinositol and phosphoinositides. Activated PI3K phosphorylates PIP2 to PIP3, which regulates numerous cellular processes such as membrane transport, metabolism, proliferation, apoptosis, and migration. Following the formation of PIP3, it binds to several proteins, including AKT, also known as protein kinase B (PKB). Serine/threonine kinase (AKT) is involved in many cellular processes, including protein synthesis, glucose metabolism, cell proliferation, and survival. A key component of the PI3K/AKT network is the serine/threonine kinase (mTOR), known as the mammalian target of rapamycin. This serine/threonine kinase (mTOR) forms two cellular complexes known as mTORC1 and mTORC2, each with different subunit compositions and substrate selectivity. Through mTOR activation, phosphorylation of ribosomal S6 kinase beta-1 (p70S6 kinase (p70S6K)), a primary target of rapamycin, occurs. Phosphorylated mTOR initiates a kinase cascade [17,36,40,43,44].

The PI3K-AKT-mTOR signaling pathway represents a highly conserved signaling system in eukaryotic cells that promotes survival, differentiation, and regulation of proliferative activity. Components of this signaling pathway are involved in transmitting signals from growth factors to transcription factors. Disruption of signaling along the PI3K-AKT-mTOR pathway can contribute to the modulation of processes such as cell death and proliferation. It is well known that the PI3K-AKT-mTOR pathway is the most frequently activated signaling cascade during the tumor transformation of eukaryotic cells and may play a role in developing resistance to anti-tumor therapies. Disruption of the components of this signaling pathway—such as increased PI3K activity, reduced PTEN function, and enhanced activity of serine-threonine protein kinase (AKT)—is a common cause of treatment resistance and tumor progression [36,40,45]. Furthermore, excessive activation of the PI3K-AKT-mTOR signaling pathway has been shown to promote epithelial–mesenchymal transition and metastasis, significantly impacting cell migration [36,40,46].

Research conducted by Xiao Q. et al. demonstrates that inhibiting the PI3K/AKT/mTOR pathway can be mediated by reducing the expression of phosphorylated PI3K (p-PI3K), phosphorylated AKT (p-AKT), and mTOR in human melanoma cell lines A375 and SK-MEL-28 under the influence of hydrogen sulfide [47].

As previously mentioned, inadequate functioning of signaling pathways contributes to the activation of melanoma cell malignancy. This process is often initiated by the activation of the RAS/RAF/MAPK signaling cascade due to mutations in *BRAF* (50%), *NRAS* (30%), or neurofibromin 1 (*NF1*, 10%), which together represent the most common driver mutations found in melanomas [5,9,17,18,39,42].

The molecular pathogenesis of skin melanoma is closely linked to the activation of the mitogen-activated protein kinase (MAPK) signaling pathway, primarily caused by mutations in the *BRAF* gene [9,39,48]. The *BRAF* gene—a proto-oncogene located on the long arm of chromosome 7 (7q34)—encodes the BRAF protein, which is involved in regulating cell proliferation. The *BRAF V600* mutation is an activating mutation in the *BRAF* gene at position 600 of exon 15, where a nucleotide coding for valine is replaced with another amino acid. The most common mutation associated with melanogenesis is the substitution of valine with glutamic acid at position 600 (*BRAF V600E*) within the kinase domain [39,49,50,51,52]. The second most prevalent mutation at this position, contributing to 10–30% of mutations, is *BRAF V600K*, where valine is replaced with lysine, followed by *BRAF V600R,* which accounts for 3–7% of mutations and involves the substitution of valine with arginine [53]. Ultimately, *BRAF* mutations lead to constitutive activation of signaling through the MAPK pathway, resulting in continuous transmission of proliferative signals to the nucleus and subsequent loss of control over tumor growth [8]. Among all melanomas, those with the *BRAF V600E* mutation exhibit extreme aggressiveness [6,7,8]. Furthermore, in patients with melanoma, there is a classification for the *BRAF V600* gene segment as “wild type,” indicating that there are no activating V600 mutations in the *BRAF* gene.

Another significant mutation involved in the development of skin melanoma is in the *NRAS* gene [15]. The *NRAS* gene is also a proto-oncogene located on the short arm of chromosome 1. Its expression products—RAS proteins—are small intracellular GTPases. Signaling through receptor tyrosine kinases (RTK) activates the *NRAS* genome segment. Active RAS GTPases can stimulate various cellular processes such as proliferation, differentiation, apoptosis, and intracellular interactions, which also occur through the MAPK pathway and PI3K cascade. Oncogenic missense mutations in codons 12, 13, or 61 lead to aberrant RAS function. Mutations in codons 12 and 13 affect the P-loop (Walker A motif) of the protein, while mutations in codon 61 disrupt the catalytic activity in the switch II domain of *NRAS* gene products [54]. As a result, melanoma cells with *NRAS* gene mutations exhibit overexpression of cyclin D1 and therefore the transition from the G1 phase to the S phase of the cell cycle [15].

The *NF1* gene, a tumor suppressor located on chromosome 17, encodes neurofibromin 1, a protein composed of over 2800 amino acids and containing multiple functional domains. One of these domains, consisting of 360 amino acids, shares significant similarity with the catalytic domain of a GTPase-activating protein. This domain is known to negatively regulate the RAS system by converting active RAS-guanosine triphosphate (RAS-GTP) into inactive RAS-guanosine diphosphate (RAS-GDP), thereby inhibiting signals from RAS pathway components [14,55,56]. Mutations in the *NF1* gene lead to reduced expression of this gene, which removes the inhibitory effect on the MAPK/RAS signaling pathway and its downstream effector pathways, resulting in their excessive activation. Consequently, this modulation affects the proliferative and apoptotic activities of tumor cells [57].

**Figure 1:**
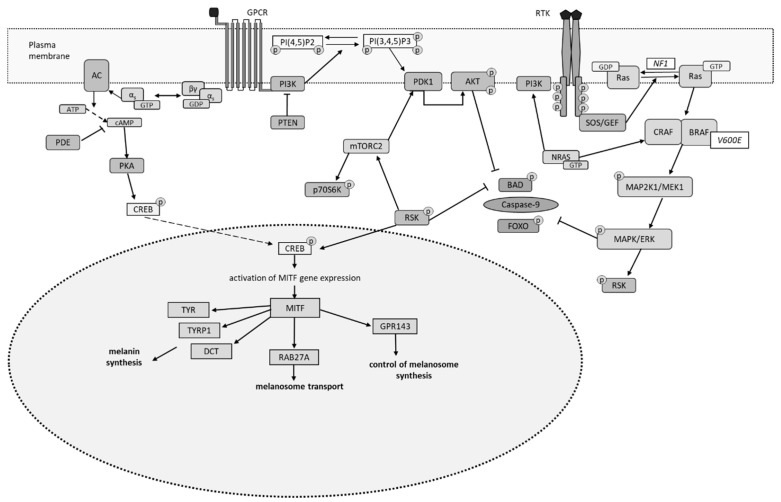
Signaling pathways and the melanin-synthesizing function of melanoma cells harboring genomic mutations [4,15,18,19,39,56,57].

Mutations in the *BRAF*, *NRAS*, and *NF1* cause hyperactivation of the RAS/MAPK signaling pathway, while mutations in the *PTEN* and *AKT* genes cause hyperactivation of the PI3K signaling pathway. Recent studies have indicated that the RAS/MAPK signaling pathway is involved in melanoma development through interactions with ligands and receptors or by engaging with other pathways such as the PI3K and cAMP signaling pathways [57]. Hyperactivation of the RAS/MAPK pathway is observed in 90% of skin melanoma cases. More than half of all skin melanomas have activating mutations in *BRAF*, while 15–20% have mutations in *NRAS*. This results in the activation of RAS/MAPK and PI3K-mTOR signaling pathways, leading to increased proliferation and dysregulation of apoptotic cell death [56]. Melanoma cells that exhibit activation of the PI3K-AKT-mTOR signaling pathway show a characteristic where the level of activation of serine/threonine kinase AKT3 is dependent on the disease stage. It has been established that 60% of sporadic melanoma cases show AKT3 activation due to amplification of the *AKT3* gene and less frequently due to mutations in *AKT3* or *PI3K* genes. 40–60% of skin melanoma cases involve inactivation of the PTEN phosphatase (a tumor suppressor). PTEN phosphatase dephosphorylates PIP3, which negatively regulates the PI3K-AKT signaling pathway. Melanomas with *NRAS* mutations do not typically exhibit reduced levels of PTEN. However, in cases with *BRAF* mutations, there is an increased expression of the *AKT3* gene segment due to amplification (Figure 1) [18].

Another mechanism that contributes to preventing tumor cell death is the upregulation of the *Olig2* gene, which encodes a protein belonging to the oligodendrocyte transcription factor family. It is a key transcription factor characterized by a basic helix-loop-helix (bHLH) structure. Normally, this protein is synthesized in nervous tissue during embryonic development. However, it has been found that this protein is overexpressed in cancer cell lines from lung carcinoma, breast cancer, and melanoma. Lee J.E. et al. investigated the role of Olig2 in apoptosis, migration, and invasion of melanoma cells. They found that Olig2 is overexpressed in melanoma cells, exerting an anti-apoptotic effect. A reduction in Olig2 levels is associated with pro-apoptotic effects due to increased levels of p53 and the activity of caspases 3 and 7. The authors also demonstrated that decreased Olig2 levels inhibit the migration and invasion of melanoma cells by suppressing epithelial–mesenchymal transition and the expression of matrix metalloproteinases (MMP-1, MMP-2, MMP-9), which are induced by transforming growth factor beta (TGF-β). Furthermore, Olig2 is involved in the later stages of the MEK/ERK and PI3K/AKT signaling pathways, which are crucial for the metastatic progression of melanoma [58].

## 4. The Role of Reactive Oxygen Species in the Regulation and Execution of Apoptosis in Tumor Cells

One theory of carcinogenesis posits that oxidative stress develops in normal melanocytes, subsequently leading to their malignant transformation. The process of a normal cell transitioning to a tumor cell involves a shift in the directionality of metabolic pathways. One mechanism underlying this influence is an alteration in the balance between pro-oxidants and antioxidants. As a result of changes in redox status, oxidative modification of macromolecules occurs, including protein components that regulate and execute cellular processes in tumor cells.

Reactive oxygen species (ROS) can be considered a universal instrument of tumor transformation, as they are capable of participating in the maintenance of the functional properties of biological membranes, intracellular redox systems, and enzyme activity [59,60]. From the perspective of metabolic regulation, two primary targets of ROS action in cells are distinguished: interaction with metal-containing proteins and oxidation of SH groups in protein molecules.

Modulation of apoptosis via ROS can occur either through the direct involvement of ROS in redox signaling via site-specific receptor sequences, which triggers a cascade of intracellular processes (protein kinase reactions, including phosphorylation of proteins such as transcription factors), or through the direct action of ROS on protein molecules, including enzymes: protein kinases, phosphatases, caspases, and transcription factors. However, in both cases, the modulation of apoptosis is fundamentally based on oxidative modification of cellular proteins.

The mechanism of sensory interaction between ROS and receptors is made possible by the presence of cysteine-rich domains. For instance, the Fas receptor is characterized by the presence of three extracellular SH sites. Furthermore, ROS can participate in the induction of apoptosis through a receptor-mediated mechanism by interacting with specialized protein complexes such as the DISC (death-inducing signaling complex), which subsequently activates caspase-8. In the case of signal transduction via the TNF receptor family, ROS act directly as secondary messengers, exerting pro-apoptotic effects [60].

The regulatory influence of ROS on apoptotic processes is mediated through changes in the functional activity of pro- and anti-apoptotic factors. To date, several dozen such redox-regulated transcription factors have been identified, including the antioxidant responsive element (ARE), NF-κB, p53, Apaf-1, nuclear factor (erythroid-derived 2)-like 2 (Nrf2), activating protein-1 transcription factor (AP-1), and others. The presence of cysteine residues in the structure of transcription factors, which serve as ROS sensors, enables their interaction within the DNA-binding domain [26,61].

The hydroxyl radical (^•^OH), generated via the Fenton reaction, can modulate the activity of MAP kinases, which play a key role in apoptosis. In tandem with hydrogen peroxide, it can act as a regulator of apoptosis through the activation of apoptosis signal-regulating kinase (ASK), JNK, and p38 MAP kinase [62]. ^•^OH-induced oxidation of the cysteine-containing site of ATP/ADP translocase, located in the inner mitochondrial membrane, converts it into a permeable, non-specific channel (pore), facilitating the release of proapoptotic mitochondrial proteins into the cytoplasm [63].

On the other hand, ROS exert an activating effect on mitochondrial proteins of the Bcl-2 family, specifically Bcl-2 and Bcl-XL, which are capable of cleaving the caspase activator Apaf-1 or caspase-8 [64]. The inhibition of apoptosis is associated with S-nitrosylation of the active site of caspases, contributing to the “escape” of cells from apoptotic death. For instance, Cys^163^ in the active site of caspase-3 acts as a trap for NO^•^, causing the enzyme to lose its catalytic activity [65,66,67].

Thus, the redox status of the cell plays a critical role in the functioning of both effector proteins and regulatory proteins of apoptosis—including enzymes, receptors, transcription factors, and others—exerting influence on their activity. Consequently, ROS can be regarded as key regulators of the apoptotic type of cell death. The accumulation of ROS and the development of oxidative stress promote oxidative modification of macromolecules, including reversible and irreversible modification of proteins.

## 5. Oxidative Modification of Proteins as a Critical Technology for Molecular Regulation of Tumor Cell Metabolism

The primary inducers of oxidative protein modification are currently considered to be ROS, variable-valence ions, lipid peroxidation products, and carbon-containing carbonyl components (malondialdehyde, ketoaldehydes, 4-hydroxy-2-nonenal, etc.) [68]. The structural features of protein molecules render them major scavengers of ROS. The presence of sulfur-containing and aromatic amino acids within the polypeptide chain enables the efficient trapping of specific ROS molecules [28].

It has now been established that the interaction between the hydroxyl radical and the α-carbon atom of the amino acid backbone plays a key role in the formation of the “radical center” of the damaged polypeptide chain, which is associated with protein fragmentation. Meanwhile, the superoxide anion radical facilitates the formation of amides and carbonyl derivatives of proteins. These derivatives are stable products formed through covalent modification involving the amino acid radicals of proline, arginine, lysine, threonine, cysteine, and histidine. One type of irreversible oxidative modification of proteins is carbonylation. Carbonylation of amino acid residues in proteins (i.e., modification at the level of the primary structure) leads to subsequent, more substantial irreversible alterations in the protein molecule. This is manifested as changes in protein functional activity, aggregation, and fragmentation of polypeptide chains exposed to ROS. Such structural damage results, among other consequences, in a sharp increase in protein susceptibility to proteolytic degradation by proteases and a loss of their functional activity [69].

Special mention should be made of the reversible oxidative modification of the polypeptide chain involving glutathione, which is formed via weak intra- and intermolecular covalent bonds with the sulfhydryl group of cysteine (glutathionylation). Glutathione and the associated enzymatic redox proteins of the thiol-disulfide system (peroxiredoxins, glutaredoxins, and thioredoxins) mediate the process of S-thiolation/de-thiolation of protein active centers, protecting them from irreversible oxidative modification and inactivation [28,29], thereby contributing to the maintenance of functional activity of both proteins and cells as a whole. Furthermore, SH-containing amino acid residues of cysteine and methionine within the polypeptide serve as targets for ROS attack. This is due to the low ionization potential of the sulfur atom in cysteine amino acid radicals [29,70].

According to several researchers, one form of oxidative stress associated with alterations in the SH-status of protein metabolism is disulfide stress. This process arises in cells under conditions of NADPH deficiency and an excess of disulfide bonds in proteins and peptides, often in the context of glucose deprivation. Proteins are potential targets for disulfide stress due to the presence of SH groups in their molecular structure. The resulting oxidative stress promotes an increase in ROS content within the tumor cell. This is accompanied by a change in the oxidation state of the sulfur atom in SH groups, rendering them reactive for the formation of disulfide bonds. Proteins involved in the execution and regulation of apoptosis, when modified at SH groups, participate in disulfidoptosis. Some researchers suggest that disulfidoptosis represents a distinct form of cell death induced by oxidative stress, contributing to various diseases, including cancer. Thus, disturbances in the thiol-disulfide system and disulfide stress play a key role in tumor progression [71].

The number of identified redox-sensitive signal transduction pathways in cells continues to grow. Currently, a prominent area of scientific inquiry involves elucidating the functional characteristics of molecular systems resulting from conformational changes in protein molecules (phosphorylation, methylation, carbonylation, ubiquitination, glutathionylation, nitrosylation, etc.). Intracellular signal transduction directly involves protein molecules, ranging from ligand–receptor interaction (the hydrophobic protein domain of the receptor) through the regulation of enzyme activation and transcription factor binding to the genome to the translation, folding, and refolding of proteins. Upon ROS action on pro- and anti-apoptotic targets—either directly or via intracellular redox-dependent signaling systems—the cell may undergo simultaneous activation of multiple, interacting molecular pathways [72,73].

Signal transduction regulation in cells is associated with changes in the redox state of thiol groups in proteins and peptides, including glutathione. Electron transport along the side chains of functional –CH_2_–SH groups of conserved cysteine residues in proteins underpins their redox sensitivity. Transcription factors that regulate cell death programs can alter their activity under the influence of components of redox-sensitive signaling systems, as well as through their own oxidative modification. Protein glutathionylation is a reversible macromolecular modification and constitutes an important regulatory mechanism in biochemical reactions [28,29]. Therefore, investigating the involvement of oxidatively modified proteins in the regulation of intracellular processes and their role in the disruption of apoptosis execution in tumor cells represents a pressing scientific endeavor.

## 6. Oxidative Modification of Proteins in Melanoma Cells

It is well established that aggressive forms of melanoma harboring mutations exhibit resistance to targeted therapy. In our view, oxidative modification of proteins may represent an alternative molecular approach for modulating components of signaling pathways to activate the apoptotic type of cell death in melanoma tumor cells.

The redox-dependent modulation of signaling pathway protein components in melanoma cells harboring mutations remains poorly understood.

Specifically, Srivastava et al. [74,75] propose the regulation of S-nitrosylation via inhibitors of NO synthase as a novel means of modulating the MEK-ERK signaling pathway in *NRAS*-mutant melanoma. The targets of S-nitrosylation in signal transduction are kinases and specific phosphatases, whereas in the execution of apoptosis, they are caspases and transcription factors. The authors suggest that by blocking S-nitrosylation, the sensitivity of *NRAS*-mutant melanoma cells to antitumor therapy can be enhanced.

In a study conducted using a mouse model of IRF7-C435-SNO melanoma, Dai et al. [76] proposed targeting the interferon signaling receptor to increase the sensitivity of melanoma cells to immune checkpoint inhibitor therapy by blocking its S-nitrosylation.

According to Carvalho et al. [77], investigating the role of redox proteins and the modulation of redox homeostasis may provide key insights for the development of novel therapeutic strategies.

## 7. Conclusions

One of the key elements in the pathogenesis of tumor growth is the dysregulation of programmed cell death in the context of oxidative stress [3,62,72,78,79]. Recent studies have focused on understanding the role of the cell’s redox status in regulating gene expression, activity of enzymes and transcription factors, intracellular apoptosis signaling, and other processes [3,21,79]. However, the molecular mechanisms underlying the dysregulation of apoptosis in pathological conditions associated with oxidative stress, including tumor growth, are still not fully understood.

Disruption in the regulation of lethal cell programs is attributed to changes in the balance between pro-apoptotic and anti-apoptotic proteins, which arise from the sequential activation of redox-dependent signal transduction elements by various factors, both intracellular and extracellular [72,79,80]. Interprotein interactions play a crucial role in assembling and functioning enzyme complexes involved in intracellular metabolism. The activity of regulatory, effector, structural, and transport molecules of polypeptide nature may be founded on conformational changes induced by endogenous and exogenous ligands, leading to dysregulation of intracellular processes, including apoptosis. Protein molecules serve as targets for regulating both intracellular signal transduction and the direction of metabolic pathways [80,81].

Since proteins collectively not only determine the functional capabilities of individual cells but also represent molecular targets for pharmacological intervention, selectively managing reversible and irreversible oxidative modifications of proteins (glutathionylation and carbonylation), including proteins involved in apoptosis as regulators and effectors, holds significant promise for developing personalized approaches to treating malignant tumors [72,78,80,81].

**Figure 2:**
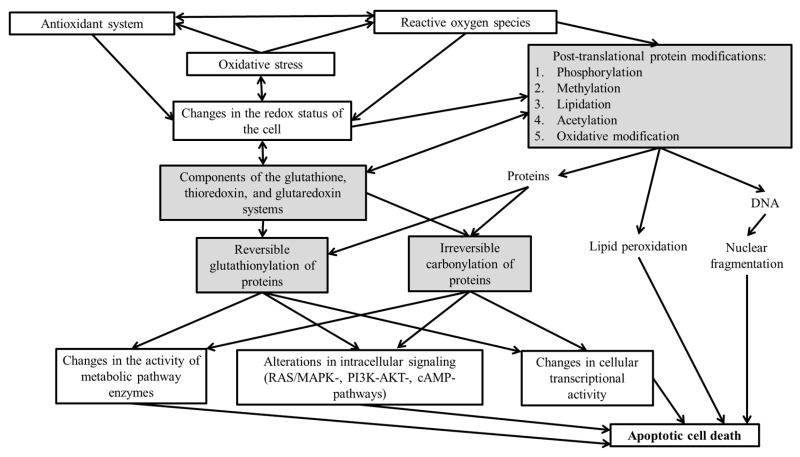
Potential molecular targets for regulating apoptosis of melanoma tumor cells [33,36,37,78]. Note—Potential molecular targets are highlighted in color.

Exploring methods to correct dysregulation of apoptosis and functions induced by oxidative stress opens up broad prospects for molecular technologies in medical practice. This could enhance the effectiveness of existing pathogenetic therapies for numerous socially significant diseases characterized by apoptotic dysregulation in the presence of oxidative stress. In this context, managing oxidative modifications of proteins can be seen as a potential molecular target for eliminating the dysregulation of apoptosis in melanoma cells (Figure 2).

Thus, the formation of a tumor cell clone in melanoma results from the uncontrolled proliferation and evasion of cell death in malignant tumor cells, driven by a combination of genetic alterations and metabolic changes (e.g., oxidative modifications of proteins), leading to neoplastic transformation and impaired response to inhibitory signals from protein molecules regulating cell growth and survival. A critical task for experimental science is to identify molecular markers for early diagnosis and therapeutic approaches for treating patients with melanoma characterized by genomic mutations. Studying the molecular mechanisms regulating metabolism, redox homeostasis, apoptosis, and tumor cell proliferation will help to identify potential targets for developing targeted therapies for skin melanoma.

## Data Availability

No new data were created or analyzed in this study. Data sharing is not applicable to this article.
